# Some New Methods of Treatment for Retroflexion of the Uterus

**Published:** 1889-10

**Authors:** 


### SOME NEW METHODS OF TREATMENT FOR RETROFLEXION OF THE UTERUS.

Retroflexion of the uterus has long been regarded as a troublesome and often incurable affection. Yet, it has never been looked upon as a dangerous disease, or one which in any way threatens the life of the patient. It must therefore prove somewhat startling to the general practitioner to learn of the radical methods of treatment for this condition which have recently come into vogue among the gynecologists. Alexander's operation of shortening the round ligaments is now no longer a new procedure. It has been done so often that it has lost the charm of novelty. Unfortunately, it is not as successful in its permanent results as was anticipated by its advocates. Anatomically the operation is a perfect one, but practically it often fails to give permanent relief. In view of the imperfection in the results of Alexander's operation, Kelly, of Philadelphia, has devised the operation of hysterorhaphy, which consists in opening the abdomen, correcting the retroflexion and stitching the fundus to the anterior abdominal wall. The results of this operation have thus far been most satisfactory. It is to be feared, however, that in case the patient should subsequently become pregnant, the fixation of the fundus might lead to troublesome consequences. Other operators have therefore modified the procedure by opening the abdomen and folding the round ligaments upon themselves, and thereby shortening them—in other words, "taking a reef" in them. Polk, of New York, accomplishes the same end by tying the round ligaments together in the median line in front of the uterus. These procedures all involve, of course, the opening of the abdomen, an operation in which the boldest operators still recognize an element of danger, although no deaths have yet been recorded from hysterorhaphy. Kelly has therefore recently performed the operation in two cases without opening the abdomen, by pushing the womb upward through the vagina, until the fundus can be felt above the pubes, and then thrusting a long needle, armed with silk, through the abdominal wall and the fundus, and bringing it out on the opposite side of the abdomen. The operation is thus shorn of the danger incident to laparotomy. Kelly admits, however, that there is some liability to inclusion of a loop of intestine in the ligature.

Such operations as these will hardly be undertaken by the general practitioner. They require a practical familiarity with pelvic anatomy, an experience in gynecological manipulation and a technical knowledge which are obtained only by long practice. They are of interest, however, as showing the advances which are taking place in this department of medicine, and the boldness with which radical and seemingly dangerous operations are undertaken for the relief of comparatively slight ailments.

